# Renin-angiotensin system blockers regulate the metabolism of isolated fat cells *in vitro*

**DOI:** 10.1590/1414-431X20165409

**Published:** 2016-07-28

**Authors:** R de O. Caminhotto, R.A.L. Sertié, S. Andreotti, A.B. Campaãa, F.B. Lima

**Affiliations:** Departamento de Fisiologia e Biofísica, Instituto de Ciências Biomédicas, Universidade de São Paulo, São Paulo, SP, Brasil

**Keywords:** Adipocytes, Lipolysis, Lipogenesis, Glucose, Renin-angiotensin system

## Abstract

Due to the presence of the renin-angiotensin system (RAS) in tissues and its specific influence on white adipose tissue, fat cells are possible targets of pharmacological RAS blockers commonly used as anti-hypertensive drugs. In the present study, we investigated the effects of different RAS blockers on fat cell metabolism, more specifically on lipolysis, lipogenesis and oxidation of energy substrates. Isolated primary adipocytes were incubated with different RAS blockers (aliskiren, captopril and losartan) *in vitro* for 24 h and lipolysis, lipogenesis and glucose oxidation capacities were determined in dose-response assays to a β-adrenergic agonist and to insulin. Although no change was found in lipolytic capacity, the RAS blockers modulated lipogenesis and glucose oxidation in a different way. While captopril decreased insulin-stimulated lipogenesis (−19% of maximal response and −60% of insulin responsiveness) due to reduced glucose derived glycerol synthesis (−19% of maximal response and 64% of insulin responsiveness), aliskiren increased insulin-stimulated glucose oxidation (+49% of maximal response and +292% of insulin responsiveness) in fat cells. Our experiments demonstrate that RAS blockers can differentially induce metabolic alterations in adipocyte metabolism, characterized by a reduction in lipogenic responsiveness or an increase in glucose oxidation. The impact of RAS blockers on adipocyte metabolism may have beneficial implications on metabolic disorders during their therapeutic use in hypertensive patients.

## Introduction

The renin-angiotensin system (RAS) is recognized as an important regulator of blood pressure and has profound influence on hydroelectrolytic homeostasis, and pharmacological blockers of RAS are significant tools in the treatment of hypertension. Although RAS blockers are not used for preventing metabolic dysfunctions in humans, data from animal models indicates that they have potential beneficial effects in obesity and diabetes (1–7).

Components of the RAS have been detected in many metabolically active tissues (such as muscle, adipose tissue, liver, and pancreas). Due to the presence of the RAS, these tissues might be targets for RAS blockers (8). Adipose tissue, which metabolism and endocrine functions are intensively involved in metabolic diseases, is especially important. Adipocytes express all the components of the RAS, including the initial substrate angiotensinogen, as well as all the enzymes required for its conversion to angiotensins and main angiotensin receptors. Adipose RAS expression is also known to be exacerbated during obesity (9).

Some effects of RAS blockers in adipocytes have already been described, such as the stimulation of adipogenesis (10,11) and modulation of endocrine properties (12,13). On the other hand, as far as we know, investigations of possible effects of RAS blockers on metabolic pathways of fat cells are lacking.

Since adipocyte metabolism greatly influences cell size and, consequently, weight management, this study aimed to investigate the possible direct effects of different RAS blockers on the main metabolic pathways of fat cells (lipolysis, lipogenesis and energy substrate oxidation).

## Material and Methods

### Fat cells isolation

The Ethical Committee for Animal Research of the Instituto de Ciências Biomédicas, Universidade de São Paulo approved the experimental procedures (#022.125.02). Twenty three male Wistar rats (8-9 weeks old, 200–250 g) from the Animal Resource Center of this institute were used in the experiments. All animals were pre-anesthetized with sodium thiopental (4 mg/100 g body weight) and decapitated. A periepididymal fat pad (1 g) was excised and minced to small pieces with scissors, and digested at 37°C in EHB buffer [Earles' salts, 25 mM HEPES (N-2-hydroxyethylpiperazine-N-2-ethanesulfonic acid) and 4% bovine serum albumin (BSA)], pH 7.4, containing collagenase type I (1.25 mg/mL) from Worthington Biochemical Corporation (USA). Fat cells were then isolated according to Rodbell (14). Cell size and number were determined as previously described (15).

### Fat cells RAS blockers treatment

Briefly, about 3.5×10^6^ fat cells were maintained in DMEM (low glucose), fetal bovine serum (1%), penicillin (100 U/mL), streptomycin (100 μg/mL). Cells were left untreated or were treated for 24 h with 1-µM of the following RAS blockers: the renin inhibitor aliskiren (Aliskiren Hemifumarate, Novartis, Italy), the ACE inhibitor captopril (Sigma, USA) and the AT_1_ receptor antagonist losartan (losartan potassium, Sigma-Fluka, USA). The dose was previously defined as non-toxic by a cell viability assay (Cell Proliferation Kit II-XTT, Roche; Supplementary Figure S1), and was above the IC_50_ for aliskiren (0.6 nM) (16) and captopril (0.021 µM) (17), and the same for losartan as successfully used in Murali et al. (18) to suppress ANG II action *in vitro*. Treatment did not exceed 24 h in order to prevent cell lysis, commonly seen in fresh isolated fat cells after longer incubations, and ensure good viability during the metabolic assays. Treated cells were then washed, suspended in 60% EHB buffer and used in the metabolic assays described below. Each assay was repeated twice and one animal represents one experimental unit (n=1).

### Measurement of lipolysis

First, 40-μL aliquots of cell suspension were transferred to microtubes (0.6 mL) and incubated in EHB buffer containing 5 mM of glucose and 0.2 mM adenosine for 30 min at 37°C, to inhibit lipolysis through G protein-coupled receptors and estimate the non-specific glycerol release. Next, 20 μL of adenosine deaminase (0.2 U/mL in EHB buffer, pH 7.45) from Sigma-Aldrich (USA) was added for 30 min at 37°C, to allow adenosine to degrade. Then, aliquots of fat cells were incubated for 60 min at 37°C with a β-adrenergic agonist ([−]-isoproterenol [+]-bitartrate salt, from Sigma-Aldrich) in increasing concentrations, to produce a dose-response curve.

The final volume was 200 μL. Reaction was stopped by transferring the tubes to an ice bath, followed by centrifugation at 5200 *g* for 5 min at 4°C to isolate the cells in the reaction medium. Glycerol release was determined using an enzymatic-colorimetric method (Free glycerol determination kit, Sigma-Aldrich) and used as an index of lipolysis rate. The results are reported in nmol·10^6^ cells^-1^·h^-1^.

### Measurement of lipogenesis

Briefly, aliquots (25 μL) of cell suspension (∼7.5×10^4^ cells) were transferred to polypropylene test tubes containing 5 μL (1850 Bq/tube) of D-[U-^14^C]-glucose (Amersham Biosciences GE Health Care, UK) and 450-μL aliquots of Krebs/Ringer/phosphate buffer, pH 7.4, with 1% BSA and 1 mM of glucose, at 37°C (previously saturated with a CO_2_ (5%)/O_2_ (95%) gas mixture) and assayed in the presence of increasing insulin concentrations (dose-response curve), until final concentrations of 25 nM. These samples were then incubated in a final volume of 500 μL for 60 min at 37°C in a water bath. The tubes had a rubber stopper, and the air inside was enriched with CO_2_ (5%)/O_2_ (95%) to preserve the buffer assay from pH oscillations. At the end of incubation, the final reaction mixture was treated with 2.5 mL Dole's reagent (isopropanol:*n*-heptane:H_2_SO_4_, 4:1:0.25) for cell lipid extraction. This mixture was vigorously agitated (three times) in vortex and decanted. From the upper (heptane) layer, 500 μL were transferred to scintillation vials and the radioactivity (from D-[U-^14^C]-glucose) incorporated into lipid extract was measured in a β-counter (1450 MicroBeta TriLux, PerkinElmer, USA). The results were reported in nmol·10^6^ cells^-1^·h^-1^.

### Measurement of fatty acids and glycerol synthesis

Another aliquot (500 µL) of the lipid extraction described above was dried out naturally after a few days of rest and incubated for 60 min in 60°C with 95% ethanol as solvent (1:1) and 40% KOH to hydrolyze the ester bounds of triacylglycerol (TAG) and separate fatty acids and glycerol. Lipid extraction of this mixture was done using Dole's reagent and processed as described above. The radioactivity incorporated in the fatty acid moiety was measured and glycerol moiety radioactivity was calculated as the difference between TAG and fatty acids values. The results are reported in nmol·10^6^ cells^-1^·h^-1^.

### Measurement of glucose oxidation

For this measurement, the same lipogenesis assay was done. At the end of incubation, 200 µL of H_2_SO_4_ 8 N was added in each assay tube, quickly followed by the capping of the assay tube with a scintillation vial containing a filter paper (4×2 cm) embedded with ethanolamine (200 µL). The assembled tubes were sealed with parafilm paper and incubated for 30 min at 37°C to adsorb the ^14^CO_2_ released from D-[U-^14^C]-glucose oxidation. The radioactivity was determined in a β-counter and results are reported in nmol·10^6^ cells^-1^·h^-1^.

### Statistical analysis

The insulin or isoproterenol dose-response curves were obtained from a nonlinear regression [log (agonist) *vs* response] using the Graphpad Prism 5 software (USA). Two-way analysis of variance (ANOVA) was used for interactions between factors in dose-response data. To compare differences between drug treatments, data from basal and insulin or isoproterenol-stimulated states were analyzed by two-way ANOVA followed by Bonferroni's *post hoc* tests (*P<0.05; **P<0.01; ***P<0.001). Data are reported as means±SE.

## Results

### RAS blockers had no effects on lipolytic rates of isolated fat cells

**Figure 1:**
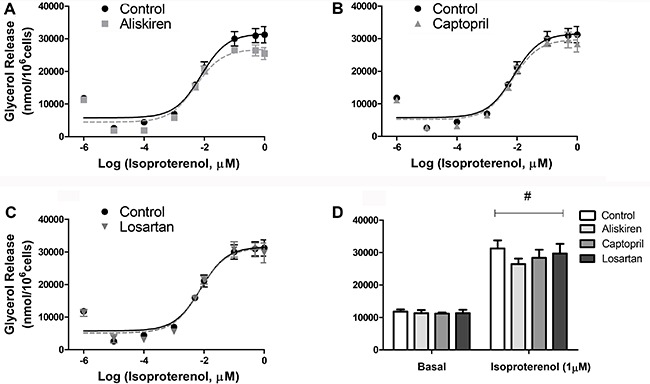
Lipolytic capacity of isolated fat cells previously treated with renin-angiotensin system blockers (*A*) aliskiren, (*B*) captopril and (*C*) losartan. Cells were incubated with increasing concentrations of isoproterenol for 60 min at 37°C and glycerol released in the medium was determined. There were no significant differences between treatments. *D*, basal and 1 µM isoproterenol stimulated lipolysis. Data are reported as the mean±SE (n=6). ^#^P<0.001 in basal *vs* 1 µM isoproterenol (two-way ANOVA).

Glycerol release from fat cells was evaluated as a marker of lipolytic rates during basal and isoproterenol-stimulated conditions (Figure 1). No effects were found after 24 h treatment with the RAS blockers.

### ACE inhibitor captopril decreased insulin-stimulated lipogenesis of isolated fat cells

**Figure 2:**
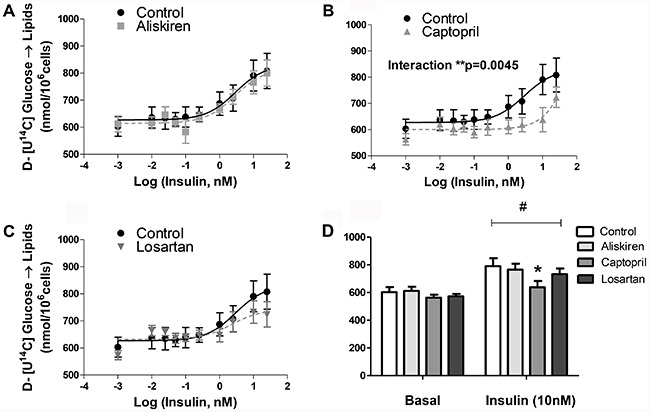
Lipid glucose incorporation of isolated fat cells previously treated with renin-angiotensin system blockers (*A*) aliskiren, (*B*) captopril and (*C*) losartan. Cells were incubated with increasing concentrations of insulin for 60 min at 37°C. D-(U-^14^C)-glucose (0.05 μCi/tube) was added to the incubation medium at the beginning of the test. At the end, adipocyte lipid content was extracted, and the radioactivity incorporated in TAG was determined. *D*, basal and 10 nM insulin-stimulated lipogenesis. *P<0.05 captopril *vs* control in 10 nM insulin-stimulated state. There was also significant lipogenic stimulus (^#^P<0.001 for basal *vs* 10 nM insulin). Data are reported as the mean±SE (n=6). Two-way ANOVA and the Bonferroni's *post hoc* tests were used.

**Figure 3:**
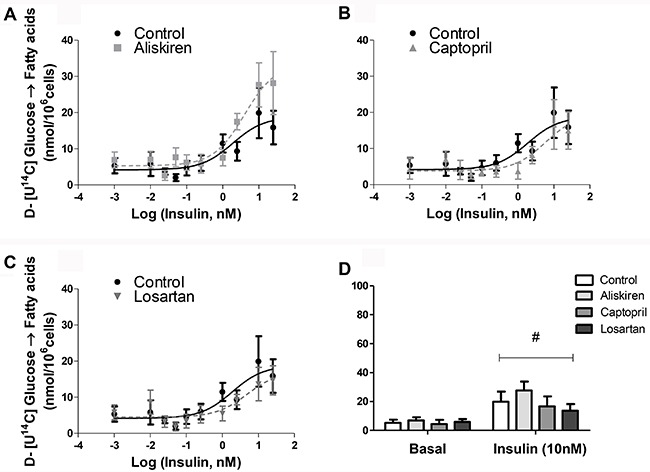
Fatty acids glucose incorporation of isolated fat cells previously treated with renin-angiotensin system blockers (*A*) aliskiren, (*B*) captopril and (*C*) losartan. There were no significant differences between treatments. Cells were incubated with increasing concentrations of insulin for 60 min at 37°C. D-[U-^14^C]-glucose (0.05 μCi/tube) was added to the incubation medium at the beginning of the test. At the end, adipocyte lipid content was extracted, and the radioactivity incorporated in fatty acids was determined. *D*, basal and 10 nM insulin-stimulated lipogenesis (^#^P<0.001 for basal *vs* 10 nM insulin) Data are reported as the mean±SE (n=6). Two-way ANOVA and the Bonferroni's *post hoc* tests were used.

**Figure 4:**
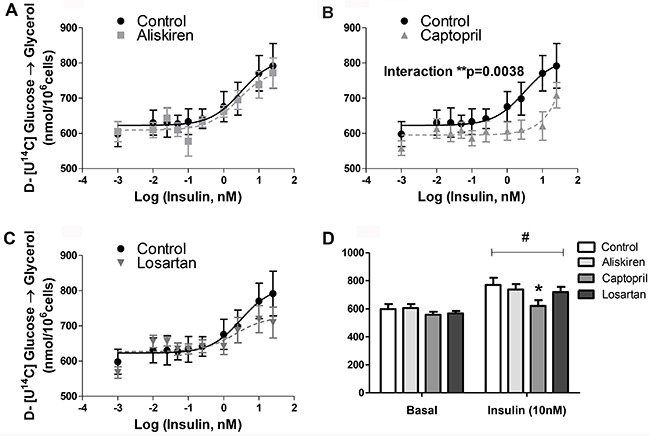
Glycerol glucose incorporation of isolated fat cells previously treated with renin-angiotensin system blockers (*A*) aliskiren, (*B*) captopril and (*C*) losartan. Cells were incubated with increasing concentrations of insulin for 60 min at 37°C. D-[U-^14^C]-glucose (0.05 μCi/tube) was added to the incubation medium at the beginning of the test. At the end, adipocyte lipid content was extracted, and the radioactivity incorporated in glycerol was determined. *D*, basal and 10 nM insulin-stimulated lipogenesis. *P<0.05 captopril *vs* control, in 10 nM insulin-stimulated state. There was also significant lipogenic stimulus (^#^P<0.001 for basal *vs* 10 nM insulin). Data are reported as the mean±SE (n=6). Two-way ANOVA and the Bonferroni's *post hoc* tests were used.

Lipid incorporation of D-[U-^14^C]-glucose was evaluated as a marker of lipogenic rates in basal and insulin-stimulated conditions. Cells treated with the ACE inhibitor captopril caused a rightward shift in the insulin-stimulated lipogenic rates in the dose-response study (Figure 2B). With 10 nM of insulin, a significantly decreased response was observed (Figure 2D), compared to the values reached by adipocytes in the control group. The other RAS blockers (renin inhibitor aliskiren and AT1 receptor antagonist losartan) did not cause any effect compared to control cells (Figure 2A, C and D). When ^14^C-glucose incorporation was analyzed separately in fatty acids and glycerol moieties after TAG hydrolysis we found that the decrease of glucose incorporation was not due to a reduction in fatty acids (Figure 3B) but in glycerol generation from glucose (Figure 4B).

### Renin inhibitor aliskiren increased insulin-stimulated glucose oxidation of isolated fat cells

**Figure 5:**
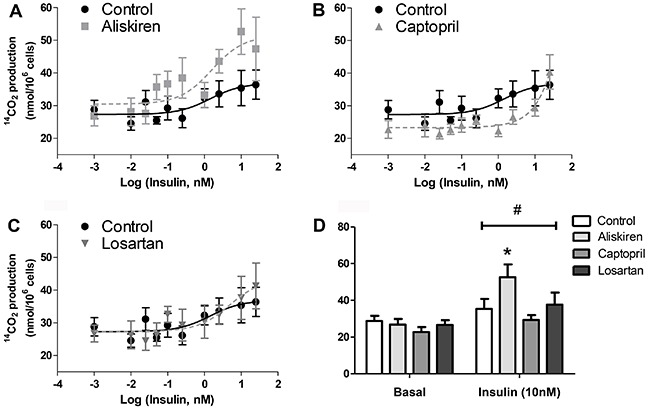
Glucose oxidation of isolated fat cells previously treated with renin-angiotensin system blockers (*A*) aliskiren, (*B*) captopril and (*C*) losartan were incubated with increasing concentrations of insulin for 60 min at 37°C. D-[U-^14^C]-glucose (0.05 μCi/tube) was added to the incubation medium at the beginning of the test. At the end, ^14^CO_2_ was collected, and the radioactivity was determined. *D*, basal and 10 nM insulin-stimulated oxidation. *P<0.05 aliskiren *vs* control in 10 nM insulin-stimulated state. There was also significant insulin glucose oxidation stimulus (^#^P<0.001 for basal *vs* 10 nM insulin). Data are reported as the mean±SE (n=6). Two-way ANOVA and the Bonferroni's *post hoc* tests were used.

The ^14^CO_2_ production was measured as a marker of D-(U-^14^C)-glucose oxidation rates in basal and insulin-stimulated conditions. Cells treated with aliskiren increased glucose oxidation rates of insulin-stimulated cells, particularly at 10 nM of insulin (Figure 5A and D), when the carbon dioxide production increased approximately 50% compared to non-treated control cells and insulin responsiveness increased approximately three times. The other RAS blockers tested, captopril and losartan, were ineffective (Figure 5B and C).

## Discussion

In this study, we demonstrated that the main metabolic aspects of fat cell function can be modulated by RAS blockers. To become hypertrophic, adipocytes need to store more energy substrates than they can mobilize or use (19).

Through lipolysis, fatty acids and glycerol are released from intracellular fat droplets in adipocytes through TAG hydrolysis (20). Some RAS products have been already associated with lipolysis activation. For example, acute exposure to ANG II decreases the spontaneous lipolytic rates in isolated human adipocytes, and the presence of AT1 receptor blocker losartan abolishes this effect, evidencing the importance of the AT1 receptor on the regulation of this process (21). On the other hand, acute exposure to ANG 1–7 increases lipolysis in isolated rat adipocytes, both in a spontaneously unstimulated and in a cathecolamine-stimulated situation, an effect that depends on the MAS receptor activation (6). However, in our study, we did not observe any degree of modulation upon lipolysis. In fact, in previous studies, the lipolytic effect of angiotensins was obtained at very high concentrations of ANG II and ANG 1-7 (6,21). Human studies with ANG II infusion at pressor-effective doses (i.e., within its biological range) did not demonstrate any significant alteration in lipolytic activity (22). Based on our model of lipolysis, the *in vitro* production of angiotensin by isolated fat cells did not demonstrate relevant effects of RAS blockers on lipolytic activity.

On the other hand, although no effect was found in the first metabolic pathway studied, a second series of experiments revealed relevant actions of RAS blockers in the lipogenic assay. Through lipogenesis, fat cells can synthesize and store lipids, particularly TAG, which is composed of three fatty acids and one glycerol molecule (20). Adipocytes treated with captopril did not normally respond to insulin, showing a lower lipogenic capacity, which was recovered only in the presence of an extremely high dose of insulin (25 nM). In addition, we observed that the intense decrease in lipogenesis induced by captopril was due to a limitation in the pathway toward glycerol synthesis, while no influence was found in fatty acids synthesis.

There are two main pathways through which glycerol can be synthesized: from glucose during glycolysis; and from glucose metabolites (mainly lactate and pyruvate) through glyceroneogenesis. In the first case, glucose is diverted from the initial phase of glycolysis, when dihydroxyacetone phosphate is converted into glycerol-3-phosphate by glycerol-3-phosphate dehydrogenase. In glyceroneogenesis, the pyruvate generated from glycolysis is then redirected back to dihydroxyacetone phosphate through the action of phosphoenol pyruvate carboxykinase (PEPCK) (20). Although PEPCK is mostly recognized as a liver gluconeogenic enzyme, its concentration in fat cells is similar as in the liver. However, adipocytes do not perform gluconeogenesis due to the lack of the two terminal enzymes which allow the reconstitution and release of glucose (23). On the other hand, this pathway is important in the production of glycerol-3-phosphate in fat cells. Indeed, glyceroneogenesis has been accounted for approximately 90% of triglyceride glycerol synthesis in rat adipose tissue (24), which reinforces our hypothesis that changes in this pathway can strongly alter glycerol synthesis in fat cells.

Considering all the above information and that captopril treatment decreased glycerol synthesis but did not alter fatty acid synthesis and glucose oxidation, we can presume that glucose, the main source of the glycerol moiety in TAG, was limited by a decreased glyceroneogenesis. In a diet-induced obesity model in mice, the treatment with ACE inhibitor prevented PEPCK increase in the liver while no effects occurred with other RAS blockers, such as aliskiren and losartan, which indicates a possible relationship between ACE inhibitors and PEPCK-related pathways, as glyceroneogenesis in adipose cells (25).

As a whole, these results help to clarify the metabolic pattern that leads to weight loss in *in vivo* studies with ACE inhibitors (3,5–7), and a possible role of ACE in weight regulation (26). Likewise, renin inhibition has also been associated with weight loss and metabolic benefits in type II diabetic (4) and diet-induced obesity models (2).

Here, aliskiren did not induce changes in the lipolytic or lipogenic capacities, which are the main metabolic pathways that control intracellular TAG content. Nevertheless, the renin inhibitor increased adipocytes capability of glucose oxidation in response to insulin, which can reflect an increase in mitochondrial function. Since impaired mitochondrial function of adipocytes occurs during the development of obesity linked to metabolic diseases, such as diabetes and insulin resistance in humans (27,28) and experimental animal models (29), our data endorse recent findings of increased metabolic activity of adipose cells in adipose-specific angiotensinogen inactivation (30). Furthermore, aliskiren treatment has shown to increase the Uncoupling Protein 1 (UCP-1), an important mitochondrial protein in white adipose tissue of type 2 diabetic KK-A(y) mice (4), which also increases mitochondrial function and energy substrates oxidation. In our experiment, we also tested whether aliskiren increases UCP-1 gene expression through real time quantitative PCR, but the result was negative (Supplementary Figure S2).

Although the tested drugs in this research showed the capacity to interfere with the RAS, a discrepancy between RAS blockers on fat cells metabolic response was found. The different effects may be explained by the drugs interference in the different steps of RAS. While aliskiren decreases the synthesis of all angiotensins (as ANG I, II, 1–7), captopril decreases only ANG II formation while it increases ANG 1–7 synthesis through the renin/ACE 2 axis (31). While ANG II is associated with obesity, oxidative stress and insulin resistance, ACE 2/ANG 1–7 axis has emerged as a beneficial antagonist of ACE/ANG II axis, which improves systemic insulin sensitivity, glucose homeostasis and body weight (32). Furthermore, ACE inhibitors can also increase bradykinin formation (31), which is emerging as another adipose tissue metabolically active peptide that decreases propensity to obesity (33).

Among the RAS blockers tested, only losartan treatment did not induce modulations in any metabolic pathway. The losartan dose tested had been successfully used in another *in vitro* study to suppress ANG II action (18). Indeed, AT1 receptors blockers become relevant only during concomitant high doses of ANG II incubation (21). Besides, although some AT1 antagonists are agonists of peroxisome proliferator-activated receptor γ (PPARγ), losartan is not recognized as a potent one (34). In 3T3-L1 adipocytes, losartan has a PPARγ agonism effect only in high concentrations (from 10 to 100 µM) (35). This possible effect of PPARγ agonism also exists in other RAS blockers (enalapril, irbesartan, telmisartan) and should be especially considered in studies where comparisons are carried out within the same step of the RAS blockade. The action demonstrated by the drugs tested herein had not yet been reported. Since PPARγ activation has an important effect in adipose tissue metabolism, the utilization of other RAS blockers, including blockers of the same step, might induce different results.

Comparisons between the action of other RAS blockers, either of the same or from different metabolic pathways, need to be done. This aspect is a limitation of our work.

In conclusion, our experiments demonstrated that RAS blockers can induce metabolic alterations in fat cells metabolism, which can help to explain the beneficial effects in obesity and weight loss in animal studies. Fat cells treated with the ACE inhibitor captopril had a limited lipogenic response to insulin, decreasing glycerol synthesis from glucose. In turn, adipocytes treated with the renin inhibitor aliskiren exhibited increased glucose oxidation in response to insulin. Drug-specific studies are encouraged, as different RAS blockers have different actions in adipocytes metabolism. Finally, the effects of RAS blockers on adipocyte metabolism may have beneficial implications on metabolic disorders during their therapeutic use in hypertensive patients, although the *in vivo* effects need to be further elucidated.

## Supplementary material

Click here to view [pdf].

## References

[1] Furuhashi M, Ura N, Takizawa H, Yoshida D, Moniwa N, Murakami H (2004). Blockade of the renin-angiotensin system decreases adipocyte size with
improvement in insulin sensitivity. J Hypertens.

[B02] Stucchi P, Cano V, Ruiz-Gayo M, Fernandez-Alfonso MS (2009). Aliskiren reduces body-weight gain, adiposity and plasma leptin during
diet-induced obesity. Br J Pharmacol.

[B03] Santos EL, de Picoli SK, da Silva ED, Batista EC, Martins PJ, D'Almeida V (2009). Long term treatment with ACE inhibitor enalapril decreases body weight
gain and increases life span in rats. Biochem Pharmacol.

[B04] Iwai M, Kanno H, Tomono Y, Inaba S, Senba I, Furuno M (2010). Direct renin inhibition improved insulin resistance and adipose tissue
dysfunction in type 2 diabetic KK-A(y) mice. J Hypertens.

[B05] De Kloet AD, Krause EG, Kim DH, Sakai RR, Seeley RJ, Woods SC (2009). The effect of angiotensin-converting enzyme inhibition using captopril
on energy balance and glucose homeostasis. Endocrinology.

[B06] Oh YB, Kim JH, Park BM, Park BH, Kim SH (2012). Captopril intake decreases body weight gain via
angiotensin-(1-7). Peptides.

[7] Dost T, Kafkas S, Gokalp F, Karul A, Birincioglu M (2014). Effects of angiotensin converting enzyme inhibition on adiponectin
levels and lipid profile in the ovariectomized-aged rats. J Pharmacol Pharmacother.

[8] Favre GA, Esnault VL, Van Obberghen E (2015). Modulation of glucose metabolism by the renin-angiotensin-aldosterone
system. Am J Physiol Endocrinol Metab.

[9] Frigolet ME, Torres N, Tovar AR (2013). The renin-angiotensin system in adipose tissue and its metabolic
consequences during obesity. J Nutr Biochem.

[10] Janke J, Engeli S, Gorzelniak K, Luft FC, Sharma AM (2002). Mature adipocytes inhibit in vitro differentiation of human
preadipocytes via angiotensin type 1 receptors. Diabetes.

[11] Janke J, Schupp M, Engeli S, Gorzelniak K, Boschmann M, Sauma L (2006). Angiotensin type 1 receptor antagonists induce human
*in-vitro* adipogenesis through peroxisome
proliferator-activated receptor-gamma activation. J Hypertens.

[12] Brody R, Peleg E, Grossman E, Sharabi Y (2009). Production and secretion of adiponectin from 3T3-L1 adipocytes:
comparison of antihypertensive drugs. Am J Hypertens.

[13] Hung WW, Hsieh TJ, Lin T, Chou PC, Hsiao PJ, Lin KD (2011). Blockade of the renin-angiotensin system ameliorates apelin production
in 3T3-L1 adipocytes. Cardiovasc Drugs Ther.

[14] Rodbell M (1964). Metabolism of isolated fat cells. I. Effects of hormones on glucose
metabolism and lipolysis. J Biol Chem.

[15] Di Girolamo M, Mendlinger S, Fertig JW (1971). A simple method to determine fat cell size and number in four
mammalian species. Am J Physiol.

[16] Wood JM, Maibaum J, Rahuel J, Grutter MG, Cohen NC, Rasetti V (2003). Structure-based design of aliskiren, a novel orally effective renin
inhibitor. Biochem Biophys Res Commun.

[17] Hooper NM, Turner AJ (1987). Isolation of two differentially glycosylated forms of
peptidyl-dipeptidase A (angiotensin converting enzyme) from pig brain: a
re-evaluation of their role in neuropeptide metabolism. Biochem J.

[18] Murali S, Zhang M, Nurse CA (2014). Angiotensin II mobilizes intracellular calcium and activates
pannexin-1 channels in rat carotid body type II cells via AT1
receptors. J Physiol.

[19] Langin D (2011). In and out: adipose tissue lipid turnover in obesity and
dyslipidemia. Cell Metab.

[20] Proenca AR, Sertie RA, Oliveira AC, Campana AB, Caminhotto RO, Chimin P (2014). New concepts in white adipose tissue physiology. Braz J Med Biol Res.

[21] Goossens GH, Blaak EE, Arner P, Saris WH, van Baak MA (2007). Angiotensin II: a hormone that affects lipid metabolism in adipose
tissue. Int J Obes.

[22] Townsend RR (2001). The effects of angiotensin-II on lipolysis in humans. Metabolism.

[23] Beale EG, Hammer RE, Antoine B, Forest C (2002). Glyceroneogenesis comes of age. FASEB J.

[24] Nye CK, Hanson RW, Kalhan SC (2008). Glyceroneogenesis is the dominant pathway for triglyceride glycerol
synthesis *in vivo* in the rat. J Biol Chem.

[25] Frantz ED, Penna-de-Carvalho A, Batista TM, Aguila MB, Mandarim-de-Lacerda CA (2014). Comparative effects of the renin-angiotensin system blockers on
nonalcoholic fatty liver disease and insulin resistance in C57BL/6
mice. Metab Syndr Relat Disord.

[26] Wang P, Holst C, Wodzig WK, Andersen MR, Astrup A, van Baak MA (2012). Circulating ACE is a predictor of weight loss maintenance not only in
overweight and obese women, but also in men. Int J Obes.

[27] Yin X, Lanza IR, Swain JM, Sarr MG, Nair KS, Jensen MD (2014). Adipocyte mitochondrial function is reduced in human obesity
independent of fat cell size. J Clin Endocrinol Metab.

[28] Dahlman I, Forsgren M, Sjogren A, Nordstrom EA, Kaaman M, Naslund E (2006). Downregulation of electron transport chain genes in visceral adipose
tissue in type 2 diabetes independent of obesity and possibly involving tumor
necrosis factor-alpha. Diabetes.

[29] Choo HJ, Kim JH, Kwon OB, Lee CS, Mun JY, Han SS (2006). Mitochondria are impaired in the adipocytes of type 2 diabetic
mice. Diabetologia.

[30] LeMieux MJ, Ramalingam L, Mynatt RL, Kalupahana NS, Kim JH, Moustaid-Moussa N (2016). Inactivation of adipose angiotensinogen reduces adipose tissue
macrophages and increases metabolic activity. Obesity.

[31] Tom B, Dendorfer A, Danser AH (2003). Bradykinin, angiotensin-(1-7), and ACE inhibitors: how do they
interact?. Int J Biochem Cell Biol.

[32] Santos SH, Andrade JM (2014). Angiotensin 1-7: a peptide for preventing and treating metabolic
syndrome. Peptides.

[33] Mori MA, Sales VM, Motta FL, Fonseca RG, Alenina N, Guadagnini D (2012). Kinin B1 receptor in adipocytes regulates glucose tolerance and
predisposition to obesity. PLoS One.

[34] Fujino M, Miura S, Kiya Y, Tominaga Y, Matsuo Y, Karnik SS (2010). A small difference in the molecular structure of angiotensin II
receptor blockers induces AT(1) receptor-dependent and -independent beneficial
effects. Hypertens Res.

[35] Schupp M, Lee LD, Frost N, Umbreen S, Schmidt B, Unger T (2006). Regulation of peroxisome proliferator-activated receptor gamma
activity by losartan metabolites. Hypertension.

